# A novel SLC25A1 inhibitor, parthenolide, suppresses the growth and stemness of liver cancer stem cells with metabolic vulnerability

**DOI:** 10.1038/s41420-023-01640-6

**Published:** 2023-09-23

**Authors:** Zhichun Zhang, Yuan Qiao, Qiuyue Sun, Liang Peng, Lichao Sun

**Affiliations:** 1 Dongzhimen Hospital, Beijing University of Chinese Medicine, Beijing, 100700 China; 2 Beijing Key Laboratory for Immune-Mediated Inflammatory Diseases, Institute of Medical Science, China-Japan Friendship Hospital, Beijing, 100029 China; 3 State Key Laboratory of Molecular Oncology, National Cancer Center/Cancer Hospital, Chinese Academy of Medical Sciences and Peking Union Medical College, Beijing, 100021 China

**Keywords:** Cancer metabolism, Cancer stem cells

## Abstract

Liver cancer stem cells (LCSCs) are recognized as key contributors to hepatocarcinogenesis, progression, and recurrence. Consequently, eradicating LCSCs has a great chance of increasing long-term survival in patients with liver cancer. Parthenolide (PTL), a natural sesquiterpene lactone product, possesses robust antitumor activity. However, the effects of PTL on LCSCs and underlying mechanisms remain unknown. Here we show that administration of PTL stimulated cell cycle arrest at the G1 phase, induced apoptosis, and decreased the stemness of LCSCs. Further research indicates that PTL caused the production of ROS and the reduction of oxidative phosphorylation (OXPHOS) and mitochondrial membrane potential (MMP) levels of LCSCs. RNA sequencing (RNA-Seq) further shows that PTL decreased SLC25A1 expression at the mRNA level and that inhibition of SLC25A1 synergistically decreased the expression of IDH2 and several pivotal genes involved in mitochondrial respiratory chain complex, resulting in the production of ROS and mitochondrial dysfunction. In addition, the inhibitory effect of PTL on mitochondrial function and self-renewal capacity of LCSCs was abolished by the knockdown of SLC25A1 or treatment with SLC25A1 inhibitor CTPI-2. Importantly, PTL prevented liver cancer growth in vivo without clearly causing toxicity. Our research shows that PTL inhibits the growth and stemness of LCSCs through SLC25A1-mediated mitochondrial function. PTL may be a potential candidate natural agent for liver cancer treatment.

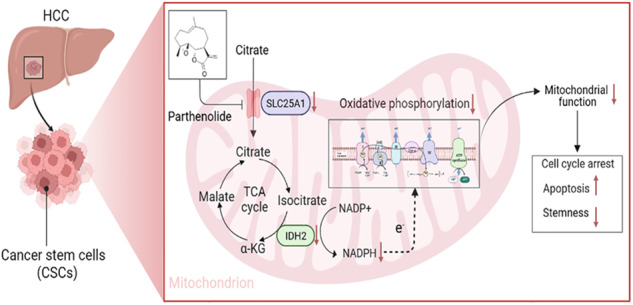

## Introduction

Primary liver cancer remains a global public health burden and challenges as it is the sixth most common morbidity and the third most lethal malignancy worldwide [1]. A significant proportion of patients with hepatocellular carcinoma (HCC) are generally asymptomatic and usually diagnosed at advanced stages, contributing to their poor prognosis [2]. Systemic therapy is still necessary for patients with advanced and metastatic liver cancer, and its pivotal role has stimulated significant efforts to exploit novel therapies [3]. However, due to resistance and systemic toxicity, current chemotherapeutics have poor clinical efficacy for advanced liver cancer [4]. Therefore, identification of novel therapeutic agents to treat liver cancer is currently of the utmost importance.

Cancer stem cells (CSCs) display unlimited potential for proliferation, differentiation, and self-renewal ability in various cancer types and account for the recurrence, metastasis, and drug resistance of cancer [5, 6]. Thus, targeting CSCs in cancer therapy brings new light to patients. Recent studies suggest that CSCs hold distinct mitochondrial properties compared to non-CSCs, as seen by their elevated mitochondrial mass and MMP and enhanced oxygen consumption rates (OCR) [7, 8]. In addition, CSCs display distinct metabolic phenotypes to non-CSCs. CSCs are more likely to increase mitochondrial oxidative phosphorylation (OXPHOS) to promote stemness properties rather than glycolysis [9–13]. Recently, therapies that directly control the mitochondria of CSCs have been introduced as potential anti-CSCs agents in preclinical investigations [14–16]. Consequently, novel therapeutic agents targeting mitochondria to eradicate CSCs need to be developed.

SLC25A1, also known as mitochondrial citrate carrier (CIC), allows the entry of cytosolic malate in exchange for the export of citrate across mitochondria [17, 18]. It has recently been linked to energy metabolism due to its ability to enhance OXPHOS to prevent cancer cells from apoptosis triggered by energy stress [19]. Moreover, SLC25A1 plays a pivotal role in regulating the redox balance and citrate pool in the mitochondria of CSCs, and its inhibition promotes the generation of ROS, thereby inhibiting the self-renewal potential of CSCs [20]. However, the roles of SLC25A1 in the abnormal energy metabolism and pathogenesis of liver cancer have not been adequately studied.

PTL, a natural sesquiterpene lactone derived from the feverfew plant (*Tanacetum parthenium*), has clinical indications including fever, migraine, and arthritis [21]. Recently, PTL has received the most attention on its anticancer effect in lung cancer [22], breast cancer [23], melanoma [24], and hepatocellular carcinoma [25], but its underlying mechanism remains unclear. Previous studies also suggest that PTL may effectively target CSCs in leukemia [26, 27] and glioma [28]. Notably, PTL has achieved a very good safety profile in Phase I/II clinical trials [29, 30]. Nevertheless, it remains unknown whether PTL has a selective inhibitory effect on liver cancer stem cells (LCSCs).

In the present study, we systematically investigated the effect and underlying mechanism of PTL on LCSCs. We revealed that PTL inhibited OXPHOS levels and the self-renewal capability of LCSCs by inhibiting SLC25A1-mediated mitochondrial function. The study puts forward a new view that regulating SLC25A1 is an encouraging therapeutic target for treating liver cancer and suggests that PTL may be a valid candidate natural agent to eliminate LCSCs.

## Results

### PTL preferentially inhibits the growth of LCSCs

**Fig. 1:**
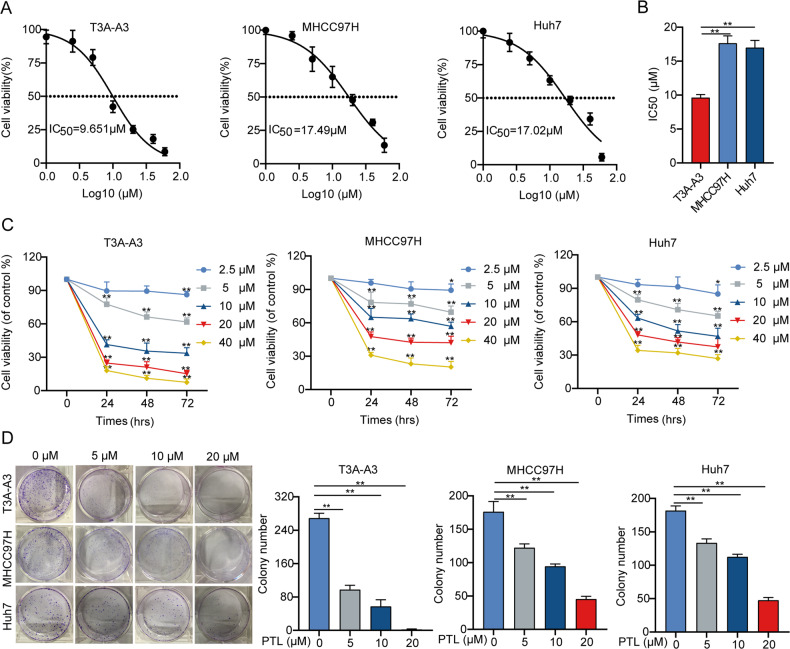
PTL inhibits cell growth in liver cancer cells. **A** The viability of T3A-A3, MHCC97H, and Huh7 cells treated with different concentrations of PTL for 24 h. **B** The IC50 of PTL in different liver cancer cells. **C** The viability of T3A-A3, MHCC97H, and Huh7 cells treated with PTL for 24 h, 48 h, and 72 h, respectively. **D** The effect of PTL on colony formation of T3A-A3, MHCC97H, and Huh7 cells.

Our previous study has shown that T3A-A3 possesses CSCs-like characteristics with significant metastasis and tumorigenicity properties and expresses a panel of LCSCs-related markers, including markers for LCSCs identification and markers for self-renewal capability [31]. Therefore, we used established T3A-A3 cell model to investigate the effects of 30 small-molecule compounds (majority of which we have shown to have inhibitory effects on hepatocellular carcinoma cell lines) on LCSCs activity in our laboratory, and selected PTL as a candidate for further investigation (Fig. S1B). Then, to confirm the inhibitory effect of PTL on the growth of LCSCs or liver cancer cells, CCK-8 assay and colony formation assay were implemented. The CCK-8 assay demonstrated that PTL dramatically inhibited the growth of T3A-A3, MHCC97H, and Huh7 cells in a dose- and time-dependent manner (Fig. 1A, C). The half-maximal inhibitory concentration (IC50) of T3A-A3, MHCC97H, and Huh7 cells at 24 h was 9.651 μM, 17.49 μM, and 17.02 μM, respectively, indicating that T3A-A3 cells were more sensitive to PTL than MHCC97H and Huh7 cells (Fig. 1B). Meanwhile, the colony formation assay further supported the finding that PTL mostly slowed the clonal growth of T3A-A3 cells (Fig. 1D). These results suggest that PTL is more likely to inhibit the growth of LCSCs.

### PTL induces the cell cycle arrest and apoptosis of LCSCs

**Fig. 2:**
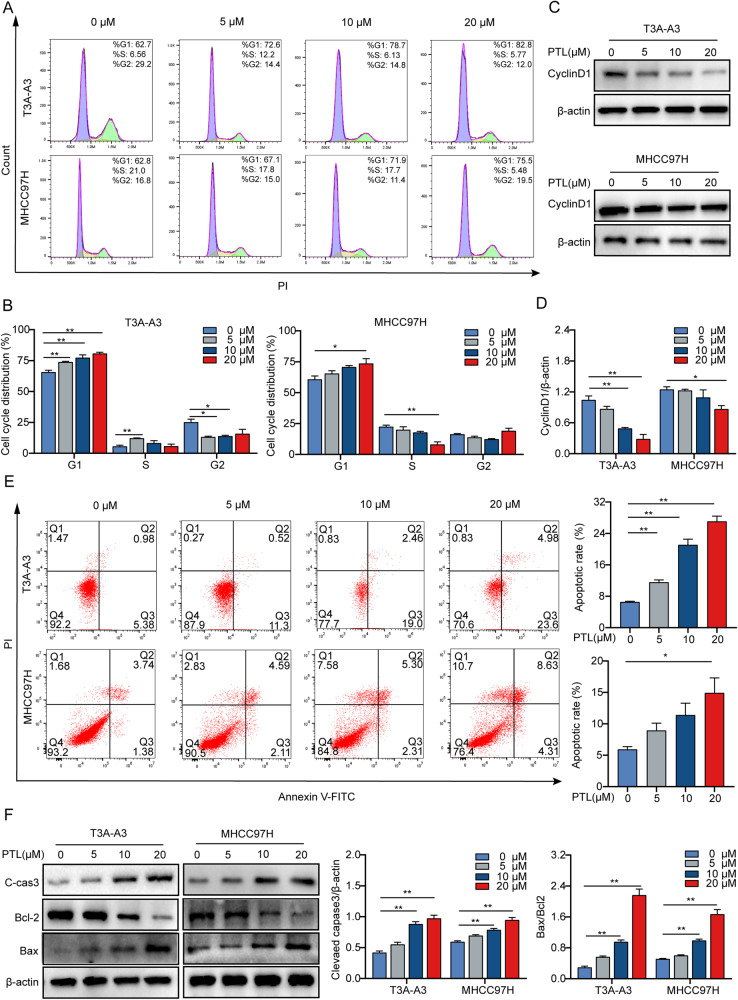
PTL induces cell cycle arrest and apoptosis of LCSCs. **A**, **B** T3A-A3 and MHCC97H cells were stimulated with PTL for 24 h, and the cell cycle was analyzed by flow cytometry. **C**, **D** The expression of cyclin D1 was detected by Western blot. **E** The apoptotic cells stained by Annexin V/PI were analyzed using flow cytometry. **F** Western blot was used to test anti- and proapoptotic proteins.

We then assessed whether the growth inhibitory effect of PTL was due to the induction of cell cycle arrest and apoptosis. Flow cytometry analysis showed that PTL dose dependently triggered cell cycle arrest at the G1 phase, the percentage of G1-phase cells in T3A-A3 and MHCC97H cells increased (Fig. 2A, B), and the reduced expression of cyclin D1 further validated that PTL-induced G1-phase arrest (Fig. 2C, D). Based on the results of the Annexin V-FITC/PI assay, PTL stimulated apoptosis of T3A-A3 and MHCC97H cells (Fig. 2E), enhanced the expression of proapoptotic proteins cleaved caspase-3 and Bax, and reduced the expression of antiapoptotic protein Bcl2, confirming that PTL triggered apoptosis of T3A-A3 and MHCC97H cells (Fig. 2F).

### PTL weakens the stemness properties of LCSCs

**Fig. 3:**
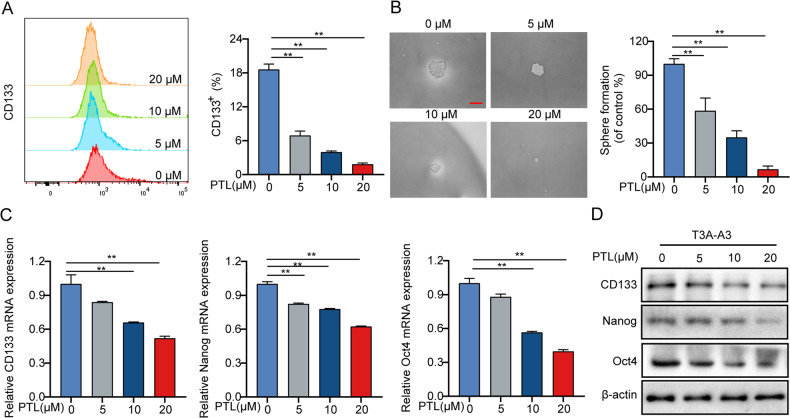
PTL weakens the stemness properties of LCSCs. **A** T3A-A3 cells were stimulated with PTL for 24 h, and CD133 expression was analyzed using flow cytometry. **B** The self-renewal ability of LCSCs was examined using sphere formation assay, scale bars, 50 μm. **C**, **D** The expression of CD133, Nanog, and Oct4 were detected by qRT-PCR and Western blot.

LCSCs exhibit high metastasis and tumorigenicity and are a key contributor to the initiation and relapse of liver cancer [32, 33]. We then investigated whether PTL treatment has an impact on the stemness of LCSCs. We initially used flow cytometry to investigate the CD133 level after PTL treatment in T3A-A3 cells. Results showed that PTL dose dependently reduced the expression of CD133 (Fig. 3A). The sphere formation assay also revealed that PTL treatment significantly reduced the number and size of spheres that formed by LCSCs (Fig. 3B). Furthermore, we found that PTL decreased the expression of a panel of LCSCs-related markers, such as markers for LCSCs identification and markers for self-renewal ability, indicating a reduced population and inhibited self-renewal ability of LCSCs following PTL treatment (Fig. 3C, D). These findings imply that PTL weakens the stemness properties of LCSCs.

### PTL impairs the mitochondrial function of LCSCs

**Fig. 4:**
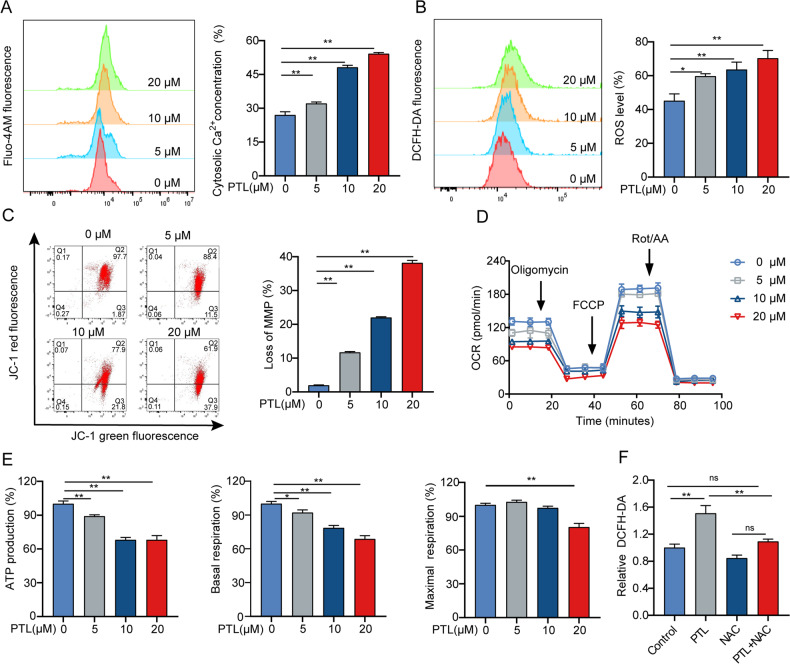
PTL impairs the mitochondrial function of LCSCs. **A** T3A-A3 cells were stimulated with PTL for 24 h, and flow cytometry was utilized to monitor Fluo-4 AM, (**B**) DCFH-DA, (**C**) and JC-1 fluorescence. **D**, **E** Seahorse analyzed the OCR. **F** Fluorescence microplate reader was utilized to examine DCFH-DA.

CSCs display distinct mitochondrial characteristics from non-CSCs [8]. They maintain stemness characteristics by exhibiting high mitochondrial function [10]. At the same time, through comprehensive regulation of redox homeostasis [34], generation of ROS [35], calcium homeostasis [36], and control of MMP [37], mitochondria regulate the energy metabolism and the gateway to cancer. Therefore, we assessed the Ca^2+^ and ROS levels after PTL treatment. We found a dramatic increase in the level of Ca^2+^ and ROS in T3A-A3 cells (Fig. 4A, B). Since MMP is one of the key sources of intracellular ROS, we also examined the effect of PTL on MMP. The results revealed that the MMP of T3A-A3 cells was dramatically decreased after PTL stimulation (Fig. 4C). Meanwhile, we found that PTL significantly reduced the OCR and inhibited basal and maximal respiration and ATP production (Fig. 4D, E). In addition, ROS scavenger NAC pretreatment partially reduced the level of ROS (Fig. 4F) in T3A-A3 cells following PTL treatment. All these results show that PTL impairs LCSCs mitochondrial function.

### PTL inhibits the expression of SLC25 A1

**Fig. 5:**
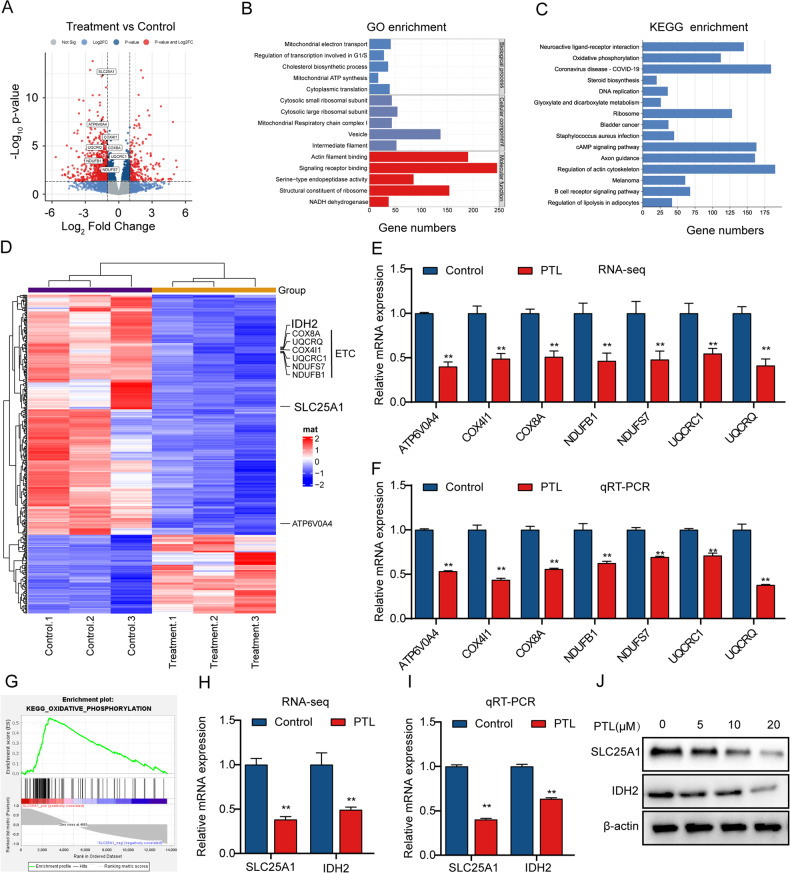
PTL inhibits the expression of SLC25A1. **A** Differentially expressed mRNAs of PTL treatment were displayed by volcano plot. **B** GO enrichment analysis of PTL treatment. **C** KEGG enrichment analysis of PTL treatment. **D** Heatmap of differentially expressed genes. Genes involved in OXPHOS pathway mRNA expression levels were determined using RNA-Seq (**E**) or qRT-PCR (**F**) assays. **G** GSEA analysis shows a positive correlation between SLC25A1 expression and the OXPHOS pathway. SLC25A1 and IDH2 mRNA expression levels were measured using RNA-Seq (**H**) or qRT-PCR (**I**) assays. **J** Western blot was utilized to analyze SLC25A1 and IDH2 protein expression levels. ETC is the abbreviation for electron transport chain.

We then utilized RNA-Seq to further examine how PTL inhibited the growth of LCSCs. We noted distinct gene expression profiles (Fig. 5A). The most significantly altered pathways of PTL treatment were linked to the mitochondrial electron transport, NADH dehydrogenase (ubiquinone) activity, mitochondrial ATP synthesis coupled proton transport, OXPHOS, and mitochondrial respiratory chain complex I (Fig. 5B, C and Fig. S2A), indicating that PTL had an impact on the mitochondria of LCSCs. Moreover, RNA-Seq and qRT-PCR analyses revealed downregulation of some crucial genes linked to the mitochondrial respiratory chain complex (Fig. 5D–F and Fig. S2B). Notably, SLC25A1 had the highest significant interaction *P* value of any mitochondrial-related gene significantly affected by PTL treatment. SLC25A1 has recently been linked to mitochondrial metabolism due to its ability to increase OXPHOS to prevent cancer cells from apoptosis triggered by energy stress [19]. A positive correlation between SLC25A1 and OXPHOS by GSEA was also found (Fig. 5G and Fig. S2C). Given that SLC25A1 is essential for mitochondrial metabolism, we investigated whether SLC25A1 is a target of PTL in LCSCs. According to the UALCAN database, SLC25A1 mRNA levels were dramatically upregulated in HCC than in normal tissues (Fig. S2D). Noticeably, we discovered that SLC25A1 mRNA levels in T3A-A3 cells were increased dramatically than in liver cancer cell lines MHCC97H and Huh7 (Fig. S2E). Meanwhile, we found consistent downregulation of IDH2 and SLC25A1 in RNA-Seq differential genes, which was supported by qRT-PCR and Western blot (Fig. 5H–J). IDH2 and SLC25A1 in liver cancer were found to have a positive Spearman correlation (Fig. S2F, *R* = 0.20, *P* = 1.931e-4). Previous studies reported that IDH2 was a downstream gene of SLC25A1 [20, 38], and played an important role in regulating the OXPHOS system [39]. Thus, we hypothesized that PTL inhibits the growth of LCSCs by inhibiting SLC25A1 and its downstream IDH2.

### PTL inhibits the growth and self-renewal ability of LCSCs by SLC25A1-mediated mitochondrial function

**Fig. 6:**
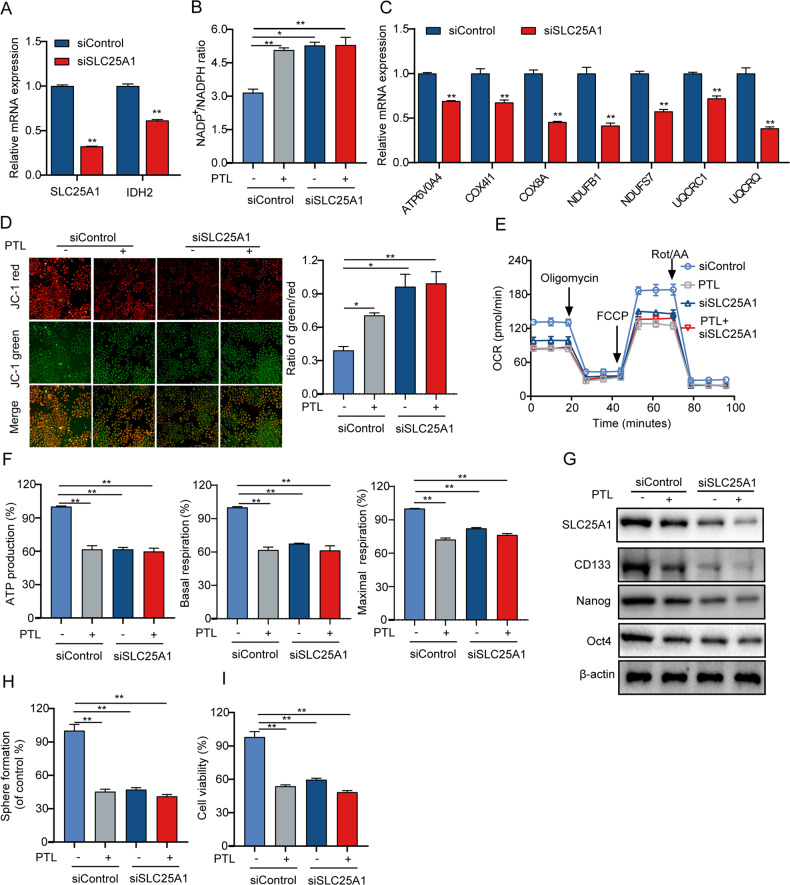
PTL inhibits the growth and self-renewal ability of LCSCs by downregulating SLC25A1-mediated mitochondrial function. **A** T3A-A3 cells transfected with SLC25A1 siRNA or control scrambled siRNA were stimulated with PTL cotreatment. qRT-PCR was then utilized to detect the mRNA expression levels of SLC25A1 and IDH2. NADP/NADPH ratio (**B**), the mRNA expression levels of genes related to OXPHOS pathway (**C**), relative MMP levels (**D**), OCR levels (**E**) relative ATP production, relative basal respiration, and relative maximal respiration (**F**), SLC25A1, CD133, Nanog, and Oct4 protein expression levels (**G**), self-renewal ability (**H**), and cell viability (**I**).

**Fig. 7:**
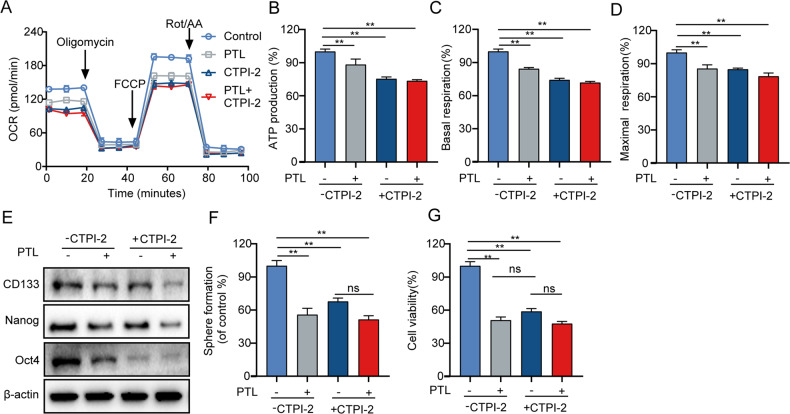
CTPI-2 affects mitochondrial function and self-renewal ability of LCSCs. T3A-A3 cells were stimulated with PTL and/or CTPI-2 for 24 h. OCR levels (**A**), relative ATP production (**B**), relative basal respiration (**C**), relative maximal respiration (**D**), the expression levels of self-renewal-related protein (**E**), the self-renewal ability (**F**), and the cell viability (**G**).

We first used the siRNA-mediated knockdown of SLC25A1 in T3A-A3 cells to verify our hypothesis. The knockdown of SLC25A1 decreased the mRNA expression of IDH2 (Fig. 6A) and increased the NADP^+^/NADPH ratio, which is consistent with the established function of IDH2 in reducing NADP^+^ to NADPH (Fig. 6B), and several genes involved in mitochondrial respiratory chain complex were also downregulated, including ATP6V0A4, COX4I1, COX8A, NDUFB1, NDUFS7, UQCRC1, and UQCRQ (Fig. 6C). Next, we investigated the effect of SLC25A1 knockout on cellular glutathione levels. As expected, the knockdown of SLC25A1 significantly reduced the level of glutathione (Fig. S2G). Furthermore, SLC25A1 knockdown significantly blocked the decrease in MMP (Fig. 6D) and OCR (Fig. 6E, F) in PTL-treated cells, indicating that the effect of PTL on mitochondrial function was dependent on SLC25A1. According to reports, SLC25A1 improved the self-renewal ability of lung CSCs and enhanced the expansion of lung CSCs [20]. Next, we investigated whether the inhibitory effect of PTL on stemness properties depended on SLC25A1. Notably, we found that the inhibitory effect of PTL on the expression of stemness-related proteins (Fig. 6G) and the self-renewal ability (Fig. 6H) of LCSCs was abolished by the knockdown of SLC25A1. Furthermore, compared to PTL treatment alone, PTL treatment in combination with SLC25A1 knockdown did not further inhibit the growth of LCSCs (Fig. 6I). The same results as SLC25A1 knockdown were obtained when treated with SLC25A1 inhibitor CTPI-2 (Fig. 7A–G). These findings support the essential role for SLC25A1-mediated mitochondrial function in the PTL-induced inhibition of growth and self-renewal.

### PTL exhibits effective antitumor activity in liver cancer xenografts

**Fig. 8:**
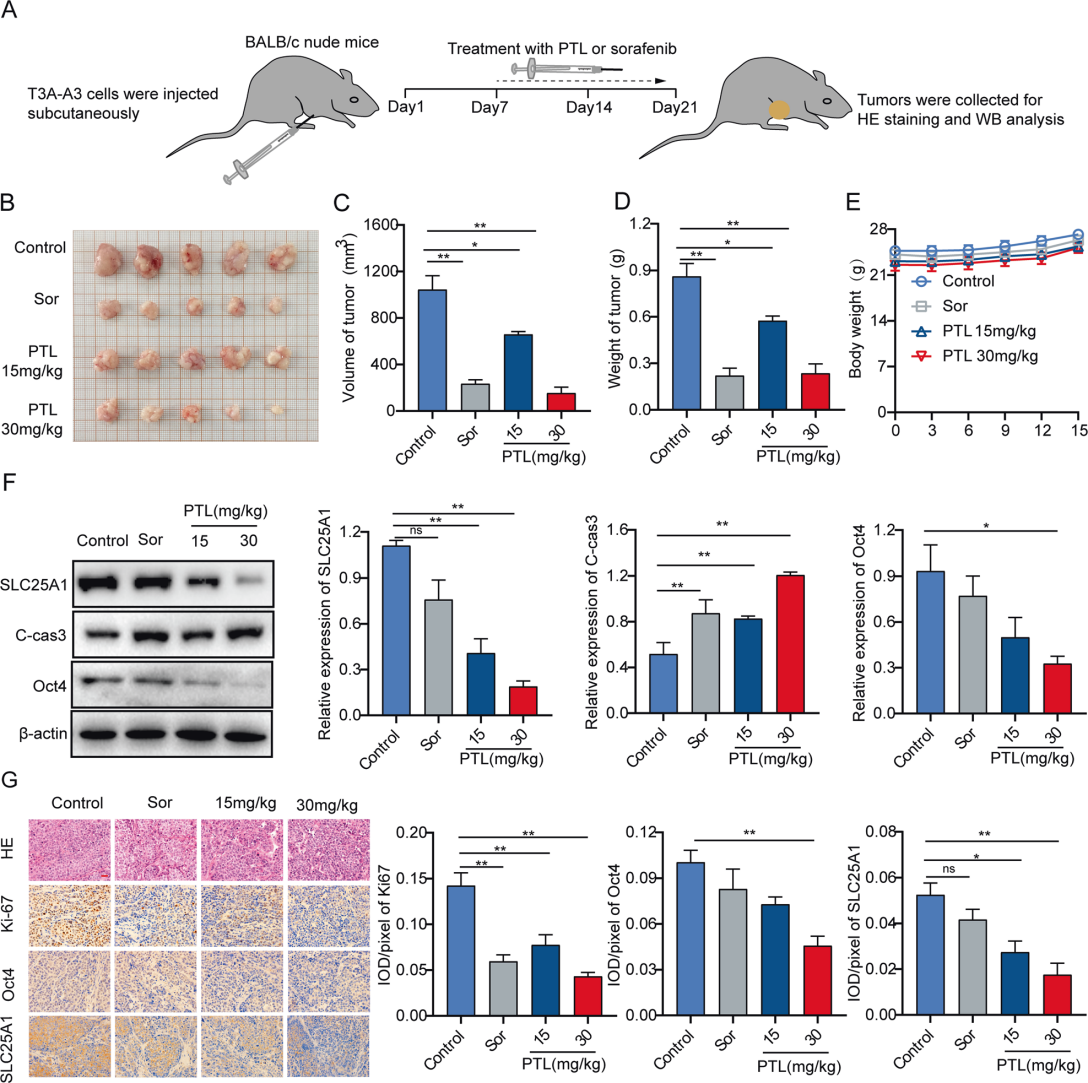
PTL exhibits effective antitumor activity in liver cancer xenografts. **A** Schematic diagram of PTL-treated mice with liver cancer xenograft tumors. **B**–**D** The effect of PTL on tumor volume and weight. **E** The effect of PTL on mice body weight. **F** The protein expressions of SLC25A1, cleaved caspase-3, and Oct4 in tumor tissues were detected using Western blot. **G** HE staining of tumor tissues and IHC of tumor tissues for Ki-67, Oct4, and SLC25A1. Scale bars, 25 μm. Sor is the abbreviation for sorafenib.

T3A-A3 cell-driven xenografts were then used to study the antitumor activity of PTL in nude mice (Fig. 8A). As expected, intraperitoneal injection of PTL inhibited tumor growth in a dose-dependent manner and significantly reduced tumor volume and weight as compared to the saline group. The inhibition of tumor growth in the high-dose group was the same as that induced by sorafenib (Fig. 8B–D). Meanwhile, mice did not display significant changes in body weight, proving that PTL did not cause obvious toxicity (Fig. 8E). In addition, Western blot and IHC analysis showed that the PTL or sorafenib treatment induced apoptosis and inhibited proliferation, as evidenced by increased expression of cleaved caspase-3 and decreased expression of Ki67. Interestingly, the expression of SLC25A1 and Oct4 in liver cancer tissue did not differ after sorafenib treatment but decreased significantly after PTL treatment (Fig. 8F, G). In conclusion, these findings demonstrate that PTL exhibits antitumor activity in liver cancer xenografts without obvious toxicity in vivo.

## Discussion

Primary liver cancer remains a global public health concern and challenge [40]. Despite significant efforts being made to develop novel drugs, existing chemotherapeutic medications have little clinical efficacy for metastatic and advanced liver cancer [4]. Natural products have outstanding value to pharmacotherapy due to their health benefits and fewer side effects, and the development of natural products for the treatment of liver cancer has drawn increasing interest [41]. Our research showed that PTL, a natural product, inhibited CSC-like properties and mitochondrial function of LCSCs. Significantly, our data show that PTL decreased the expression level of SLC25A1 and that its inhibition resulted in decreased OXPHOS and accumulation of ROS, thereby inhibiting the mitochondrial function and the self-renewal capability of LCSCs.

Recent studies have revealed that CSCs display unlimited potential of self-renewal and differentiation, which may account for clinical treatment failure [5, 6]. A growing body of evidence demonstrates that CSCs also promote the growth of HCC, and CSCs are the root of recurrence and resistance [32, 33]. Therefore, novel drugs that target CSCs can significantly improve patient outcomes and lower the likelihood of tumor relapse. Based on a continuous understanding of the biological characteristics of CSCs, various novel drugs that target CSCs have been exploited and have successfully emerged in clinical investigations, and several drugs have been approved by regulatory authorities for various purposes [42, 43]. In addition, prior research also suggested that PTL might effectively target CSCs in leukemia [27] and glioma [28], but its effect on LCSCs remains unknown. In this study, we ascertained that LCSCs were more responsive to PTL than other liver cancer cell lines. Indeed, PTL suppressed the expression of a panel of LCSC-related markers and tumor sphere formation, indicating that PTL could be an effective candidate for LCSCs-targeted therapy.

Increasing evidence indicates that CSCs display distinct mitochondrial characteristics from non-CSCs, such as CSCs exhibiting an enhanced mitochondrial mass, number, and MMP, as well as increased OCR and high expression of enzymes involved in OXPHOS [7, 8, 44]. Furthermore, CSCs display different metabolic properties when compared to non-CSCs. CSCs are inclined to produce energy through mitochondrial respiration [13, 45], and some crucial genes responsible for enhanced OXPHOS of CSCs have been identified [9, 10]. Notably, mitochondrial function plays a crucial role in CSCs functions including stemness and resistance [46]. As an alternative to existing CSCs-eradicating drugs, therapies that directly regulate mitochondrial function of CSCs have recently entered in preclinical studies [47]. In this study, we found that the most dramatically altered pathways of PTL treatment were linked to OXPHOS, mitochondrial electron transport, mitochondrial ATP synthesis coupled proton transport, mitochondrial respiratory chain complex I, and NADH dehydrogenase (ubiquinone) activity, and this suggests that PTL treatment had an impact on the mitochondria of LCSCs. Furthermore, we found that PTL-treated LCSCs had much lower MMP and OCR levels and significantly higher Ca^2+^ and ROS levels. Therefore, we hypothesized that the inhibition of cell growth by PTL might be due to its inhibition of mitochondrial function.

The solute carrier family 25 (SLC25), a large family of nuclear-encoded transporters, is composed of the products of 53 human genes [18]. The members of this superfamily transport solute through mitochondria’s inner membrane for various metabolic pathways [48]. Mutations in the SLC25 genes have recently been proven to be the cause of various diseases, highlighting the importance of SLC25 in metabolism [18]. The solute family member SLC25A1 is the only known human mitochondrial citrate carrier and transfers metabolic flux away from mitochondria [17]. SLC25A1 has been extensively investigated in relation to cancer, and abnormal expression of SLC25A1 promotes the growth and invasion of cancer cells through metabolic regulation, making it a potentially effective treatment [49]. Using the UALCAN database, we found that SLC25A1 mRNA levels were dramatically upregulated in HCC than in normal tissues and that LCSCs had significantly higher levels of SLC25A1 expression than liver cancer cell lines. Previous research has demonstrated that SLC25A1 increased OXPHOS to prevent cancer cells from apoptosis triggered by energy stress [19]. We found that SLC25A1 knockdown synergistically decreased the expression of IDH2 and some genes involved in the mitochondrial respiratory chain complex. SLC25A1 knockdown or treatment with SLC25A1 inhibitor CTPI-2 significantly blocked the decrease in OCR in PTL-treated cells, indicating that PTL’s effect on mitochondrial metabolism was mediated by SLC25A1. In addition, other studies have demonstrated that SLC25A1 promoted the growth and self-renewal of CSCs [20]. We found that SLC25A1 knockdown or treatment with the SLC25A1 inhibitor CTPI-2 abolished the inhibitory effect of PTL on stemness-related protein expression and self-renewal ability of LCSCs, suggesting that the inhibitory effect of PTL on stemness properties was dependent on SLC25A1. Furthermore, SLC25A1 knockdown in combination with PTL treatment did not further inhibit the growth of LCSCs as compared to PTL treatment alone. Therefore, we believe that PTL might inhibit SLC25A1 expression and that its inhibition resulted in reducing the expression of IDH2, thereby inhibiting the mitochondrial function and the self-renewal capability of LCSCs.

Our study found that PTL inhibited LCSCs growth by targeting on their mitochondrial function without producing obvious toxicity, which is consistent with other similar studies [23], indicating that therapeutic strategies focusing on CSCs mitochondria hold promise in enhancing long-term survival in patients with cancer [14, 15]. Sorafenib is the first-line treatment for advanced liver cancer [50]. Therefore, we used sorafenib as a positive control in in vivo animal experiments. However, only approximately 30% of patients are responsive to sorafenib treatment, and these patients tend to acquire resistance within 6 months [51]. CSCs are recognized as one of the key contributors to drug resistance. Sorafenib’s effect on CSCs is controversial. Some studies reported that sorafenib can significantly enrich CSCs [52, 53], and can also inhibit characteristics of CSCs [54, 55]. Our study found that sorafenib has no significant effect on stemness of LCSCs while influencing tumor proliferation and apoptosis.

## Conclusion

In conclusion, our research substantiates that PTL inhibited mitochondrial function and self-renewal capability of LCSCs by inhibiting SLC25A1. Our findings confirm that mitochondrial regulation of LCSCs offers a promising therapeutic approach for enhancing patient outcomes and that PTL may be a candidate natural agent for liver cancer treatment.

## Materials and methods

### Reagents

Parthenolide (PTL, 20554-84-1, purity ≥98%, Fig. S1A) was purchased from ChemFaces (Wuhan, China). N-acetyl-L-cysteine (NAC, HY-B0215) and CTPI-2 (HY-123986) were received from MCE (Monmouth Junction, NJ, USA). Antibodies against CD133 (66666-1-Ig), Nanog (14295-1-AP), SLC25A1 (15235-1-AP), and OCT4 (11263-1-AP) were all from Proteintech (Wuhan, China); β-actin (bs-0061R and bsm-33036M), Bcl-2 (bsm-52304R), and Bax (bs-0127M) were purchased from Beijing Biosynthesis Biotechnology Co., Ltd. (Beijing, China). Cyclin D1 (55506) and cleaved caspase-3 (9961) were provided by Cell Signaling Technology (Danvers, MA, USA).

### Cell culture and transfection

Liver cancer cells MHCC97H and Huh7 were propagated in Dulbecco’s Modified Eagle Medium containing 10% fetal bovine serum and 1% penicillin-streptomycin. Cancer stem cells (T3A-A3) were isolated from microvascular endothelial cells of human liver cancer and cultured as previously described [31, 56, 57]. For SLC25A1 silencing, SLC25A1 siRNA or control scrambled siRNA was transfected into T3A-A3 cells using Lipofectamine™ RNAiMAX reagent (Thermo Fisher Scientific, Loughborough, UK).

### CCK-8 assay

The viability of T3A-A3, MHCC97H, and Huh7 cells were determined by CCK-8 kit. Briefly, after cells was stimulated by PTL, CCK-8 reagent was then added and incubated at 37 °C for 2 h, and then the absorbance of each well at 450 nm was measured using a microplate reader.

### Colony formation assay

1 × 10^3^ cells/well were seeded in six-well plates and stimulated with the indicated concentrations of PTL for 24 h followed by 2 weeks to form colonies. The colonies were fixed and stained and then counted by ImageJ software (NIH, Bethesda, MD, USA).

### Sphere formation assay

Ultralow attachment plates (Corning, Shanghai, China) were used to plate 1 × 10^3^ cells/well, which were then suspended in serum-free DMEM-F12 containing 2 mM glutamine, 5 mg/ml insulin, B27 (1: 50), 2 mg/ml heparin, 20 ng/ml human epidermal growth factor (hEGF), 1% streptomycin-penicillin, 20 ng/ml basic fibroblast growth factor (bFGF), and 0.5 mg/ml hydrocortisone. Spheres were cultured for 14 days.

### Apoptosis and cell cycle analysis

Apoptosis and cell cycle were determined by the Annexin V-FITC/PI kit (BD Biosciences, San Jose, CA, USA) and the Cell Cycle and Apoptosis Analysis Kit (Beyotime Biotechnology, Shanghai, China) and analyzed by flow cytometry. FlowJo (Ashland, OR, USA) was used to analyze apoptotic cells and cell cycle distribution.

### Measurement of reactive oxygen species (ROS) generation, Ca2+ concentrations and mitochondrial membrane potentials (MMP)

The intracellular ROS, Ca^2+^ concentrations, and MMP were assessed by fluorescent probes DCFH-DA, Fluo-4/AM, and JC-1. The detailed procedure followed the manufacturer’s protocol. We also used an oxidation-insensitive dye carboxy-DCFDA probe to measure ROS. A SynergyH1 microplate reader and flow cytometry were used to measure the fluorescence intensity of each well. % of Ca2 + , ROS and MMP were calculated by Ca^2+^ (%) = (Fluo-4/AM subset / Freq. of Parent) × 100%, ROS (%) = (DCFH-DA subset / Freq. of Parent) × 100%, and MMP (%) = (JC-1 subset / Freq. of Parent) × 100%.

### NADP + /NADPH ratio and Glutathione (GSH) level

The NADP^+^/NADPH assay kit was used to measure NADP^+^/NADPH ratio (Beyotime Biotechnology, Shanghai, China). Levels of reduced GSH were determined by using the GSH assay kit (Jiancheng Bioengineering institute, Nanjing, China). The manufacturer’s instructions were carefully followed, and the absorbance of each well was measured. The NADP^+^/NADPH ratio was calculated by NADP^+^/NADPH = (NADP_total_ − NADPH)/NADPH formula.

### Oxygen consumption rate (OCR)

An XF24 extracellular flux analyzer (Seahorse Bioscience) was utilized to monitor the OCR. The detailed procedure followed the manufacturer’s instructions. Briefly, following baseline measurements, each well was sequentially injected with 1.5 µM oligomycin, 2 µM Carbonyl cyanide-4 (trifluoromethoxy) Phenylhydrazone (FCCP), and 0.5 µM rotenone/antimycin A (Rot/AA) and prepared in the assay medium and OCR was measured.

### RNA sequencing (RNA-Seq)

RNA was extracted from T3A-A3 cells using TRIzol reagent (Invitrogen, CA, USA). RNA-Seq was completed by Shanghai OE Biotech. Co., Ltd. (Oebiotech, Shanghai, China) on the IIIumina NovaSeq 6000 platforms. Differentially expressed genes were analyzed by Gene Set Enrichment Analysis (GSEA), Gene Ontology (GO), and Kyoto Encyclopedia of Genes and Genomes (KEGG) pathway enrichment analysis on the Oebiotech platform. Fold change ≥ 2.0 and *P*-value < 0.05 were the screening criteria for differentially expressed mRNAs.

### Mouse xenograft assays

The animal ethics committee of China–Japan Friendship Hospital Clinical Research gave its approval to all animal procedures. Male BALB/c nude mice (4–5 weeks old) were obtained from Beijing HFK Biotechnology Co. Ltd. (Beijing, China). T3A-A3 cells (5 × 10^5^) were subcutaneously implanted into the armpits of mice. The mice-bearing tumors were randomly assigned to four groups when they had a palpable subcutaneous tumor: control group, 10 mg/kg sorafenib group, 15 mg/kg PTL group, and 30 mg/kg PTL group. For the treatment group, 10 mg/kg sorafenib or 15 mg/kg PTL or 30 mg/kg PTL was intraperitoneally injected every day for 14 days. For the control group, an equal volume of saline was injected. Tumors were measured three times per week using calipers until a terminal event endpoint (tumor volume = 2000 mm^3^) was reached. Finally, the tumors were weighed and collected for Western blot analysis and immunohistochemistry (IHC) staining.

### Histology and IHC

IHC staining was conducted as described [31]. Tumor tissue sections were stained with hematoxylin for histological examination (HE). IHC staining was conducted with antibodies against SLC25A1, Oct4, and Ki67 and stained with the appropriate secondary antibody.

### Western blotting

The detailed procedure was as previously described [58]. The primary antibodies were shown as β-actin, CD133, Bcl-2, OCT4, Bax, Nanog, cleaved caspase-3, cyclin D1, and SLC25A1. The membranes were blocked and then incubated with primary antibodies overnight at 4 °C, followed by incubation with corresponding secondary antibodies for 2 h at 37 °C.

### Quantitative real-time PCR (qRT-PCR)

Total RNA was extracted with TRIzol reagent. The mRNA expression levels were detected by qRT-PCR using the TransScript First-Strand cDNA Synthesis SuperMix (TransGen), according to the instructions. Supplementary Table 1 lists primer sequences.

### CD133-positive cell analysis

Cells that were CD133 positive were analyzed using flow cytometry. Briefly, after the cells were stimulated by PTL, CD133/1(AC133)-PE or a negative control immunoglobulin G (IgG) isotype (Miltenyi Biotec, Teterow, Germany) was added, and the mixture was incubated at 4 °C for 10 min in the dark.

### Statistical analysis

Mean ± SEM was used to present all values, and GraphPad 8.0 software (San Diego, CA) was used to conduct statistical analysis. Unpaired t-tests were used for two groups, and one-way ANOVA test was used for groups of three or more. The Tukey’s post hoc test was conducted only if one-way ANOVA results showed a significant different with *P* < 0.05. *P < 0.05, **P < 0.01, ns, no significant difference (*P* ≥ 0.05).

### Supplementary information

Supplementary FIGURE LEGENDS

Supplementary table

Supplementary figure S1

Supplementary figure S2

Original Data File

## Data Availability

The datasets generated and/or analyzed in the current study are available from the corresponding author upon reasonable request.
