# Supplementary material for: A novel SLC25A1 inhibitor, parthenolide, suppresses the growth and stemness of liver cancer stem cells with metabolic vulnerability
Source: Cell Death Discov. 2023 Sep 23;9:350. doi: 10.1038/s41420-023-01640-6 (PMC10518014; doi:10.1038/s41420-023-01640-6)
Supplement: Supplementary file 1 — Supplementary FIGURE LEGENDS [file 41420_2023_1640_MOESM1_ESM.docx]

**Fig. S1 PTL is selected as a potential agent. A** Chemical structure of parthenolide. **B** Effects of 30 small molecular compounds on cell activity of T3A-A3 cells.

**Fig. S2 PTL affects mitochondrial-related pathways.** **A** GSEA was used to identify mitochondrial-related pathways after PTL treatment. **B** PTL treatment affected mitochondrial respiratory chain complex-related genes. **C** GSEA analysis shows a positive correlation between SLC25A1 high expression and OXPHOS by the TCGA LIHC database. **D** SLC25A1 expression in liver cancer and normal tissues in TCGA data. **E** SLC25A1 expression in T3A-A3, MHCC97H, and Huh7 cells. **F** Spearman correlation analysis of SLC25A1 expression with IDH2 expression. **G** Effects of PTL treatment and SLC25A1 knockout on GSH levels.
