# Supplementary material for: A novel SLC25A1 inhibitor, parthenolide, suppresses the growth and stemness of liver cancer stem cells with metabolic vulnerability
Source: Cell Death Discov. 2023 Sep 23;9:350. doi: 10.1038/s41420-023-01640-6 (PMC10518014; doi:10.1038/s41420-023-01640-6)
Supplement: Supplementary file 2 — Supplementary table [file 41420_2023_1640_MOESM2_ESM.docx]

**Supporting information for**

**Article**

**A novel SLC25A1 inhibitor, Parthenolide, suppresses the growth and stemness of liver cancer stem cells with metabolic vulnerability**

**Table S1. The specific primers for RT-PCR**

| **Gene** | **Forward** | **Reverse** |
| --- | --- | --- |
| SLC25A1 | 5'‑CCCCATGGAGACCATCAAG-3' | 5'- CCTGGTACGTCCCCTTCAG-3' |
| CD133 | 5′-CCTTGTGGCAAAGCTCAACC-3′ | 5′-TCACCTCCTCTCTCACCCAG-3′ |
| Nanog | 5′-CAATGGTGTGACGCAGAAGG-3′ | 5′-GCAGAGATTCCTCTCCACAGT-3′ |
| Oct4 | 5′-CGACCATCTGCCGCTTTGAG-3′ | 5′-CCCCCTGTCCCCCATTCCTA-3′ |
| ATP6V0A4 | 5′- CTGCCGAGGAAACGTGTACTT -3′ | 5′-GGCTCGAAACCCATCACAGA-3′ |
| COX4I1 | 5′- CAGGGTATTTAGCCTAGTTGGC-3′ | 5′-GCCGATCCATATAAGCTGGGA-3′ |
| COX8A | 5′-GCCAAGATCCATTCGTTGCC-3′ | 5′-CTCTGGCCTCCTGTAGGTCT-3′ |
| NDUFB1 | 5′- GTCCCTATGGGATTTGTCATTGG-3′ | 5′- CAGTTAGCCGTTCATCACTCTT-3′ |
| NDUFS7 | 5′-AGGCACGAGGTGTCCATCAGAG-3′ | 5′-CAGTTGACGAGGTCATCCAGCT-3′ |
| UQCRC1 | 5′-TGTCTCGTGCAGACTTGACC-3′ | 5′- GGCGAGGTCTAACAGTTGCT-3′ |
| UQCRQ | 5′- CGCGAGTTTGGGAATCTGAC-3′ | 5′- TGGATCTCTCGAACTCTTCAGT-3′ |
| IDH2 | 5′-CCTGCTCGTTCGCTCTCC-3′ | 5′-GCTTCGCCACCTTGATCCT-3′ |
| GAPDH | 5′-GGCACCGTCAAGGCTGAGAAC-3′ | 5′-CATGGTGGTGAAGACGCCAGTG-3′ |
