# Supplementary figures and images for: A novel SLC25A1 inhibitor, parthenolide, suppresses the growth and stemness of liver cancer stem cells with metabolic vulnerability
Source: Cell Death Discov. 2023 Sep 23;9:350. doi: 10.1038/s41420-023-01640-6 (PMC10518014; doi:10.1038/s41420-023-01640-6)

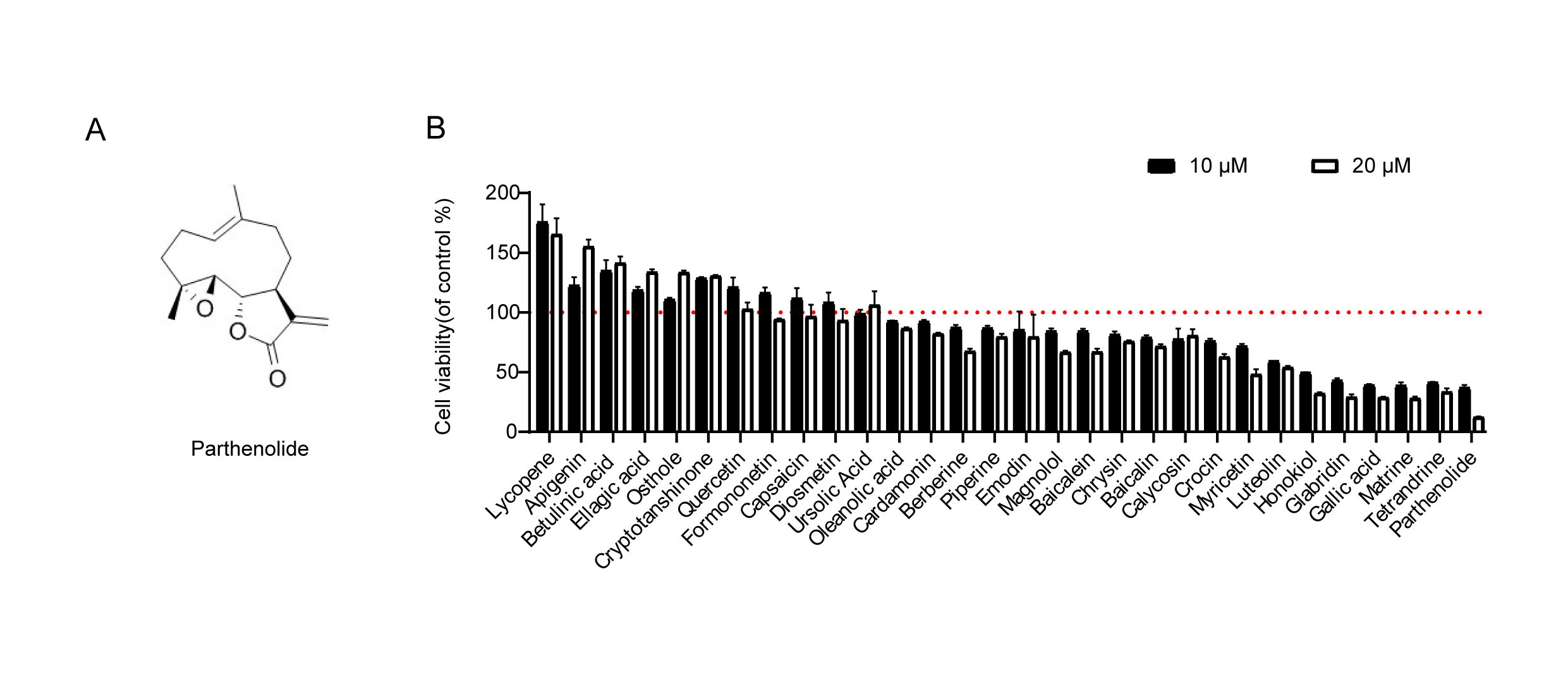

Supplement: Supplementary file 3 — Supplementary figure S1 [file 41420_2023_1640_MOESM3_ESM.tif]

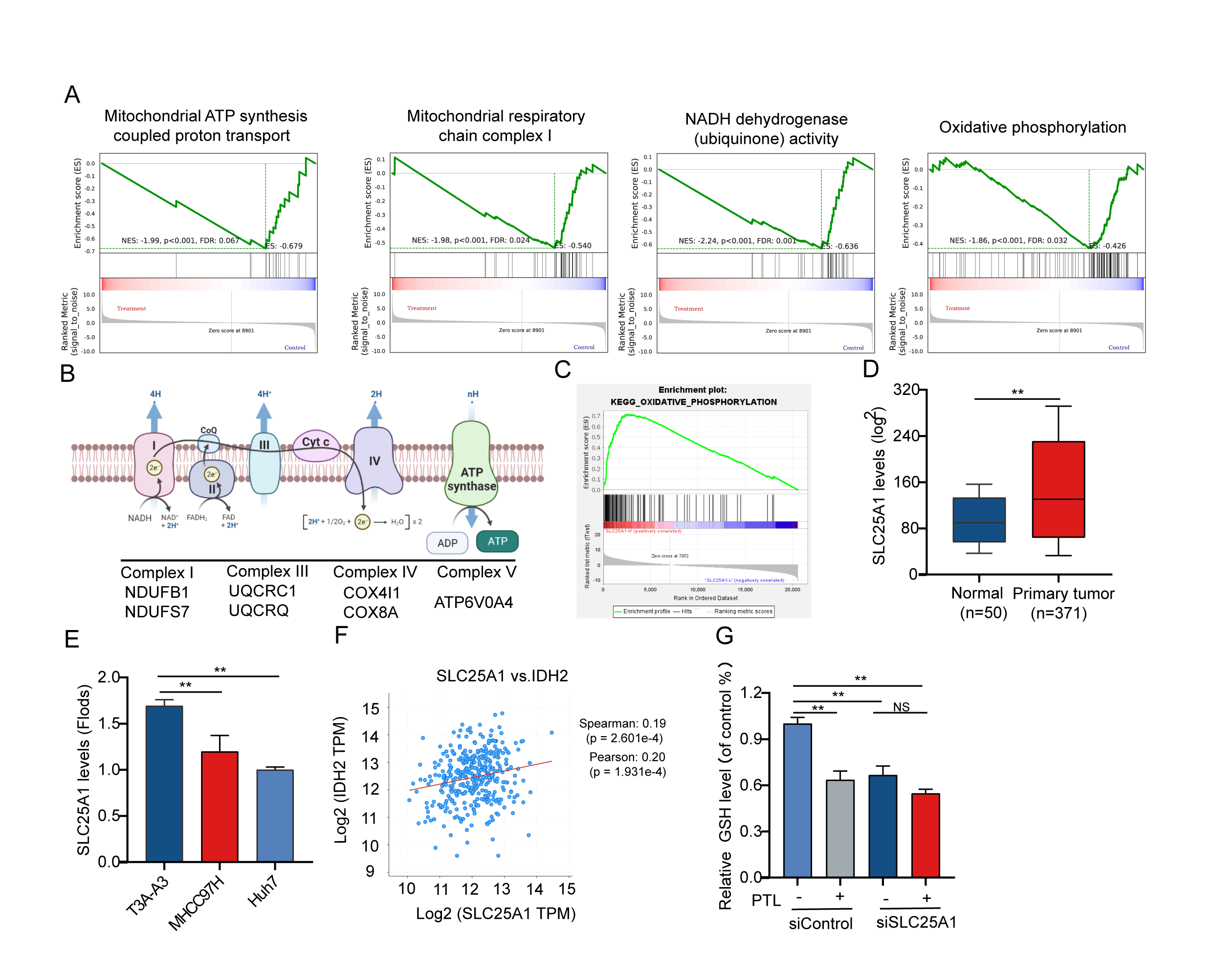

Supplement: Supplementary file 4 — Supplementary figure S2 [file 41420_2023_1640_MOESM4_ESM.tif]
