# Supplementary material for: A novel SLC25A1 inhibitor, parthenolide, suppresses the growth and stemness of liver cancer stem cells with metabolic vulnerability
Source: Cell Death Discov. 2023 Sep 23;9:350. doi: 10.1038/s41420-023-01640-6 (PMC10518014; doi:10.1038/s41420-023-01640-6)
Supplement: Supplementary file 5 — Original Data File [file 41420_2023_1640_MOESM5_ESM.pdf]

Figure 2

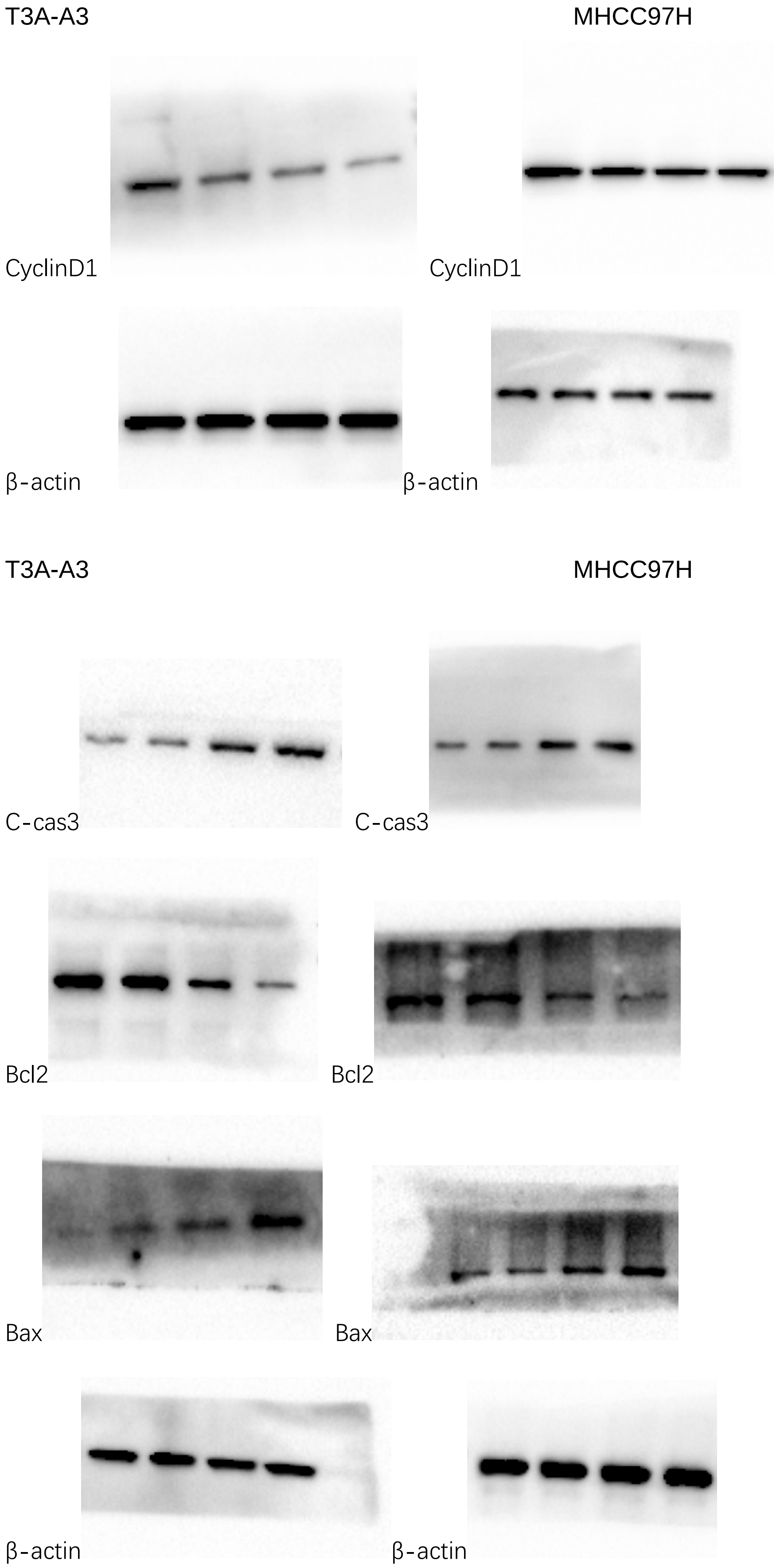

T3A-A3
MHCC97H
CyclinD1
CyclinD1
β-actin
β-actin
T3A-A3
MHCC97H
C-cas3
C-cas3
Bcl2
Bcl2
Bax
Bax
β-actin
β-actin

Figure 3

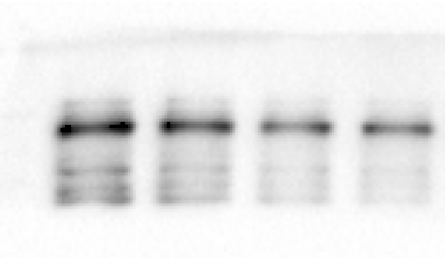

CD133

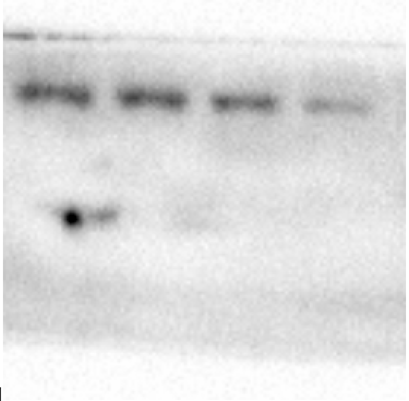

Nanog

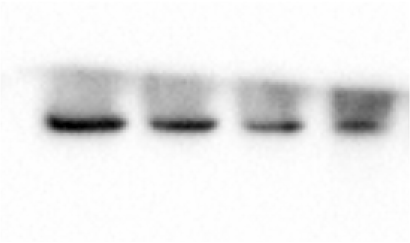

Oct4

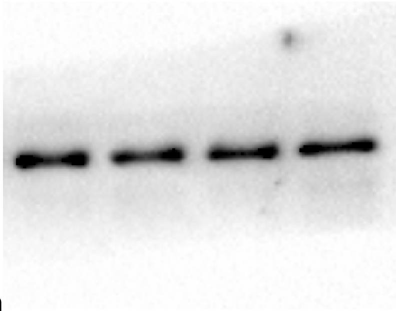

β-actin

Figure 5

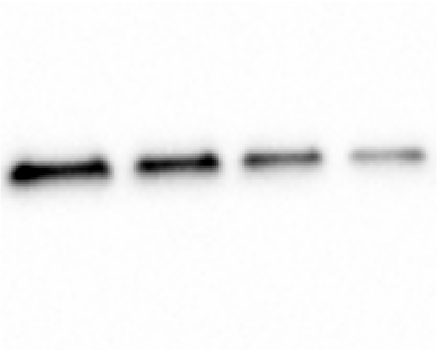

SLC25A1

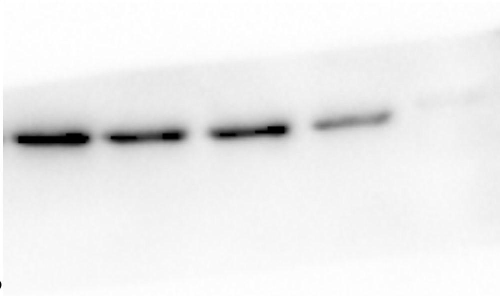

IDH2

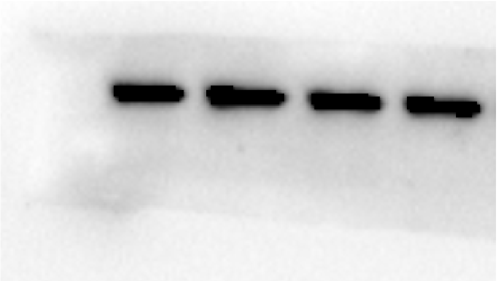

β-actin

Figure 6

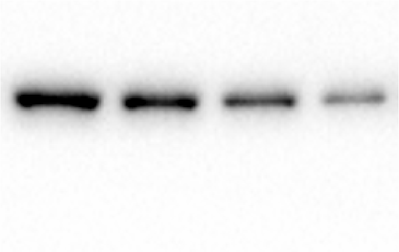

SLC25A1

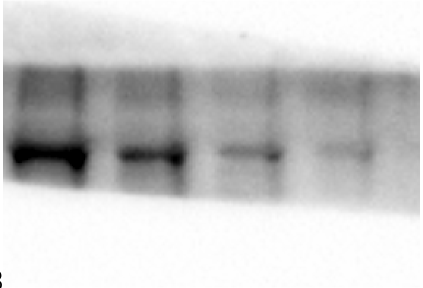

CD133

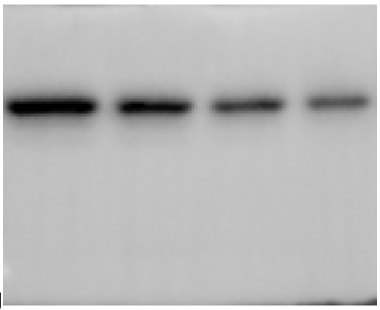

Nanog

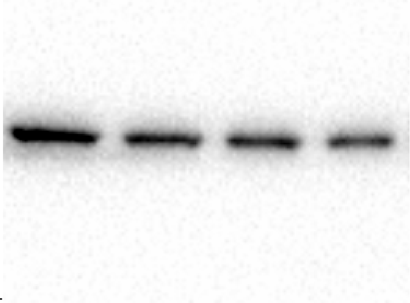

Oct4

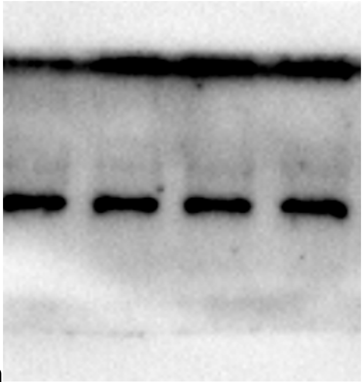

β-actin

Figure 7

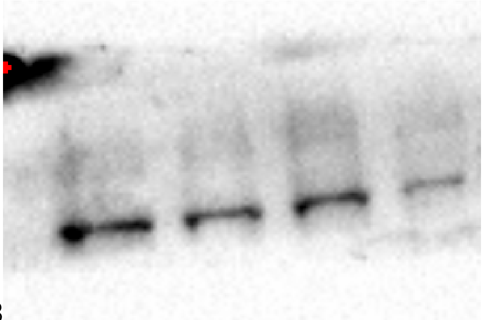

CD133

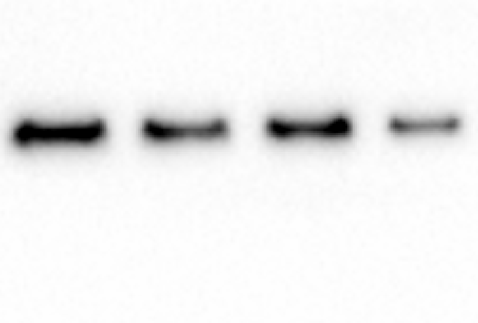

Nanog

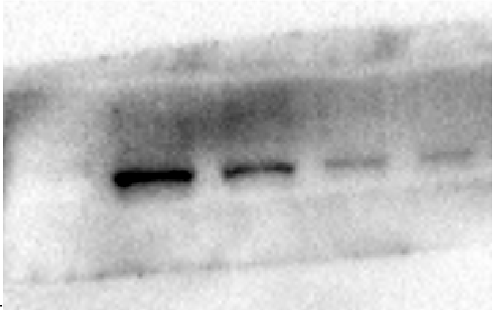

Oct4

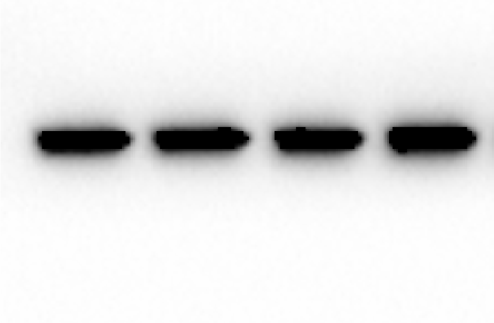

β-actin

Figure 8

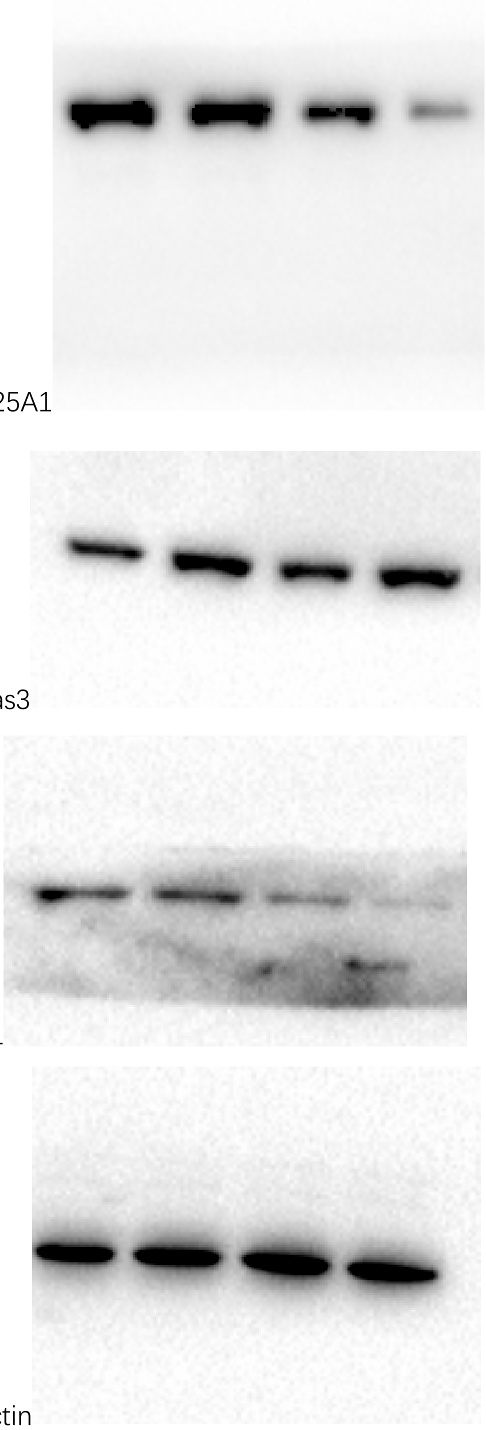

SLC25A1

C-cas3

Oct4

β-actin
